# Supplementary material for: Interplay of cis and trans mechanisms driving transcription factor binding and gene expression evolution
Source: Nat Commun. 2017 Oct 23;8:1092. doi: 10.1038/s41467-017-01037-x (PMC5653656; doi:10.1038/s41467-017-01037-x)
Supplement: Supplementary file 1 — Supplementary Information [file 41467_2017_1037_MOESM1_ESM.pdf]

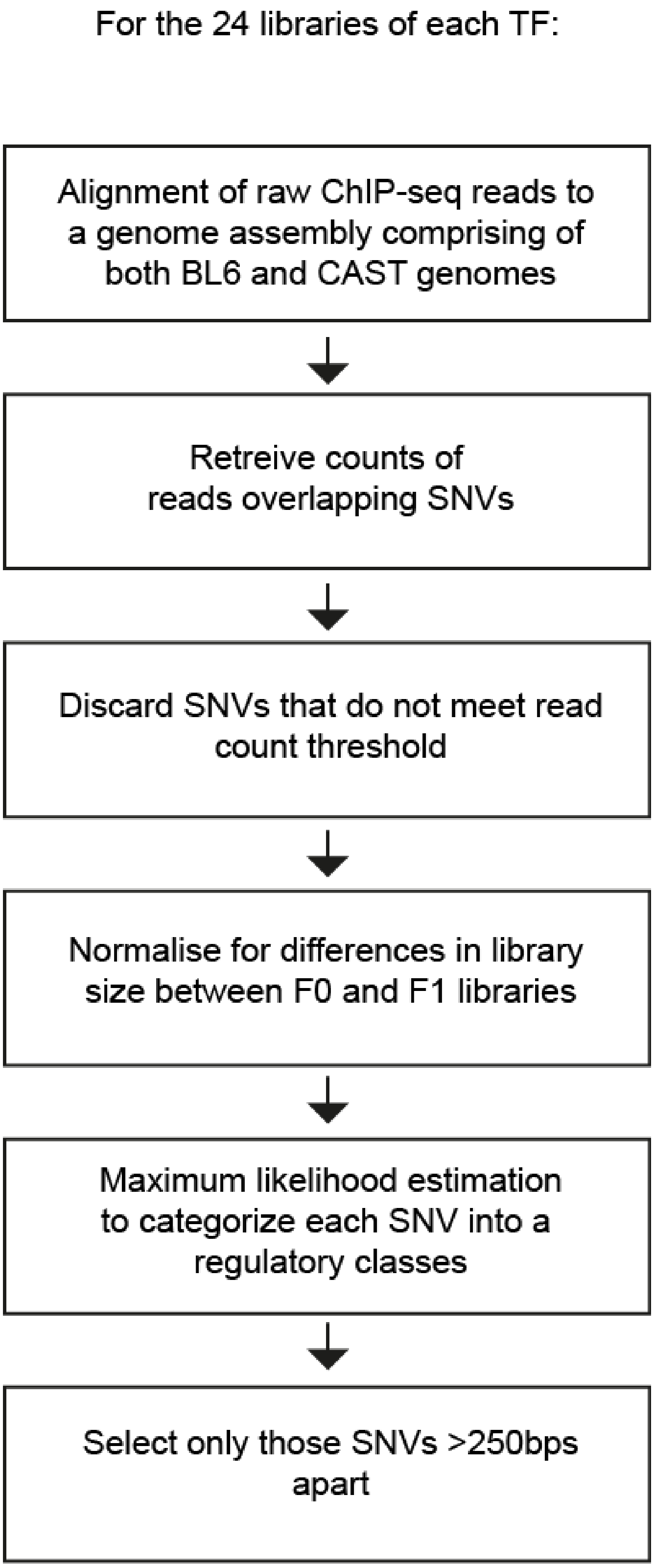

Supplementary Figure 1. Overview of computational analysis pipeline

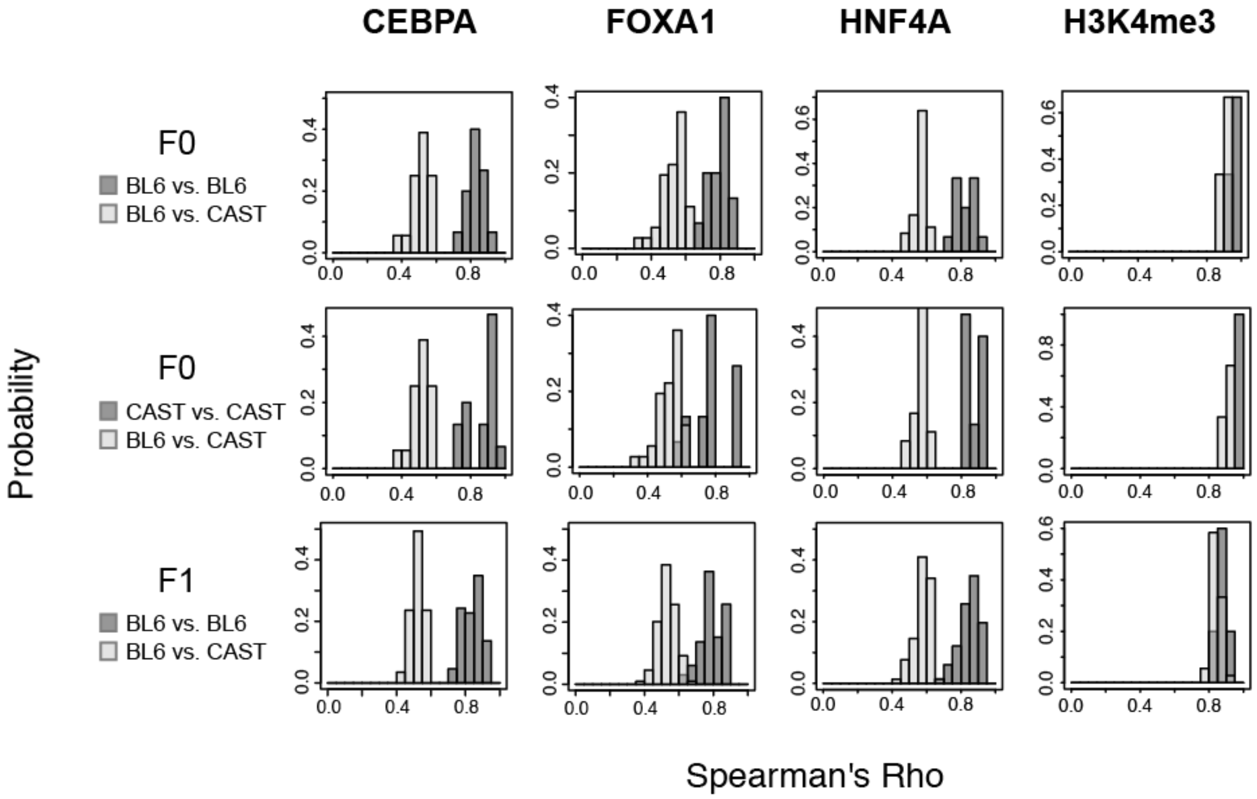

Supplementary Figure 2. Correlation of ChIP-seq measurements at SNVs between libraries of the same genetic background are consistently greater than between libraries from different genetic backgrounds. The histograms show the frequency of Spearman's Rho for ChIP-seq measurements at SVNs between libraries. For each F0 library of the same TF or histone mark, Spearman's Rho was calculated using libraries generated from the same genetic background (either BL6 or CAST) (dark color). The frequencies of these values were displayed in the same plot as Rho values generated from comparisons with libraries from individuals of the other strain (light color). For F1 individuals, correlations were made on an allele-specific basis.

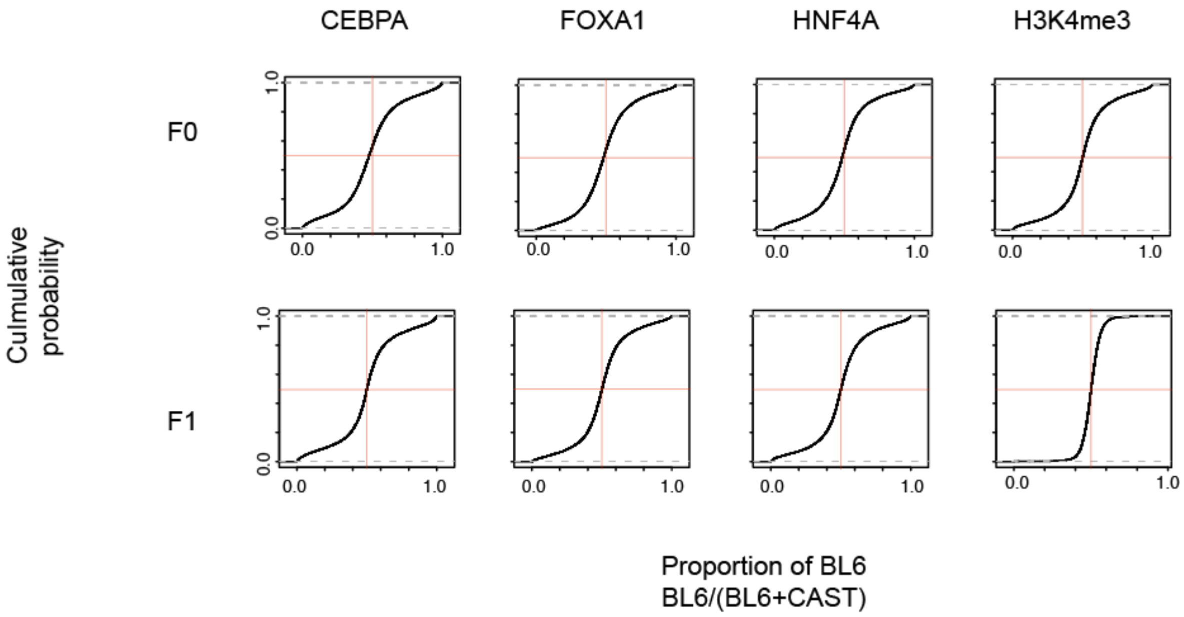

Supplementary Figure 3. A similar proportion of BL6 and CAST reads were mapped. Culmulative probability based on the ratio of BL6:CAST ChIP-seq measurements at SNVs were plotted. The vertical red line indicates 0.5 culmulative probability and the horizontal red line indicates where BL6/(BL6+CAST) = 0.5 (i.e. an equal number of BL6 and CAST reads).

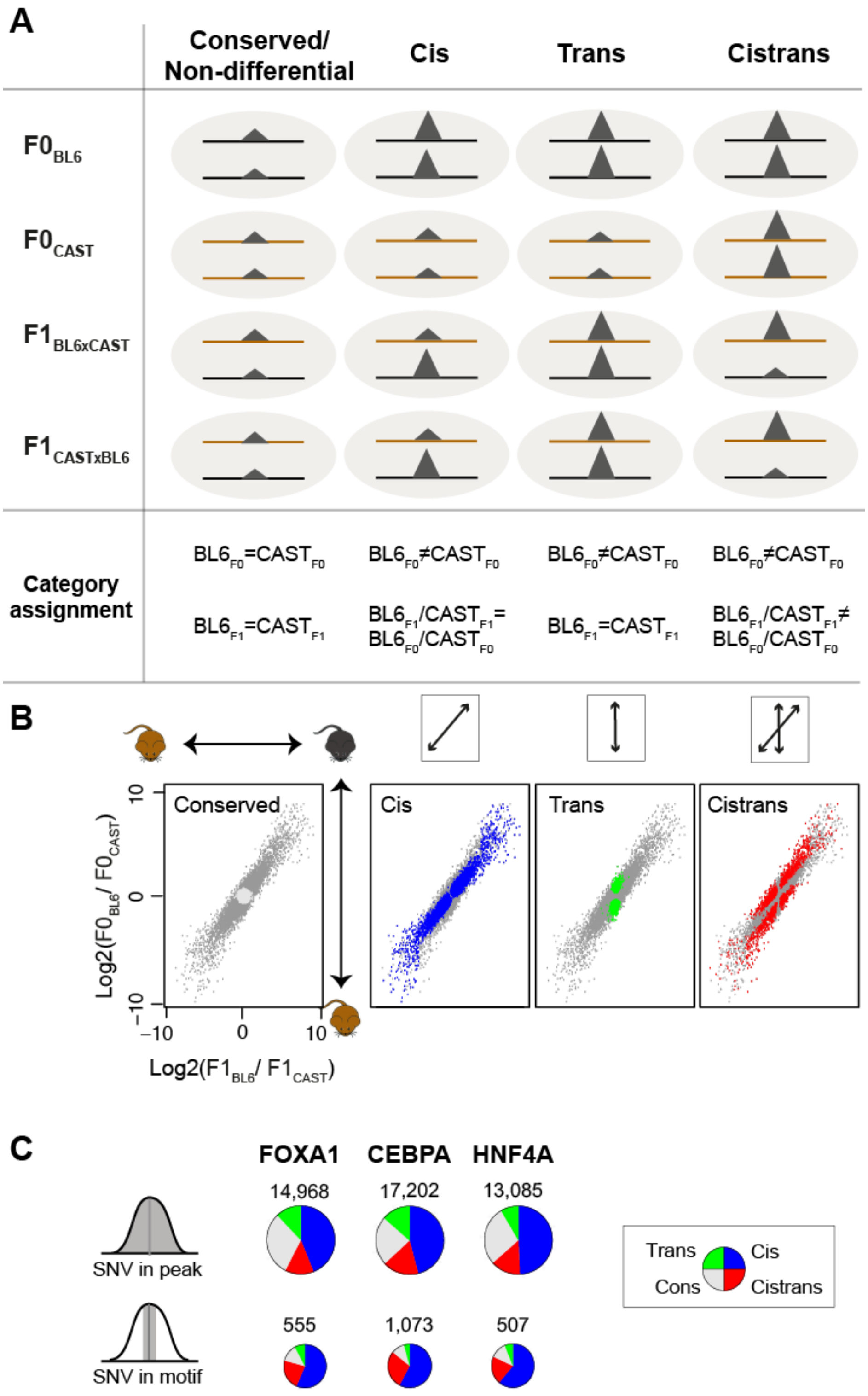

Supplementary Figure 4. The assignment of regulatory status shows that TF occupancy levels are cis-driven for a large proportion of TFBSs

(A) Regulatory categories for variation in TF binding intensities were assigned based on comparison of normalized ChIP-seq read counts between BL6 and CAST at SNVs overlapping TFBSs. Due to a common nuclear environment, trans effects that are mediated by diffusible elements are expected to impact both alleles equally. Based on this, by

comparing BL6 and CAST ratios between F0 and F1 individuals one can classify TFBSs into various regulatory categories, namely – conserved, cis, trans, and cistrans. **(B)** Scatterplots of BL6 vs CAST ratios of TF binding intensities in F0 and F1 individuals. Each point represents a separate SNV. Regulatory categories are highlighted in separate scatterplots. Dark grey color shows the remaining binding variants that do not belong in the highlighted category. CEBPA data is shown. **(C)** Pie charts depict the relative proportion of interrogated SNVs of each regulatory class for all SNVs overlapping TF bound locations, and only for SNVs positioned in the regulatory motif.

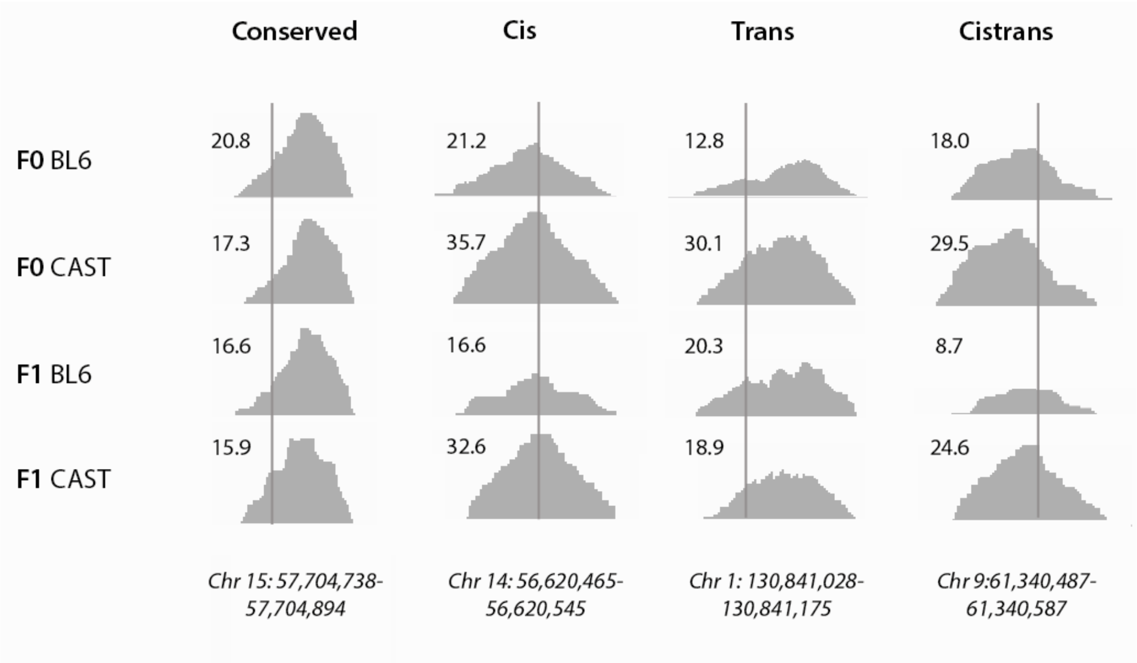

Supplementary Figure 5. Examples of CEBPA binding sites classified into different regulatory modes. TFBSs were classified based on the statistical models described (see Methods). Numbers shown are normalized ChIP-seq counts at SNV locations. These sites are marked by a vertical line.

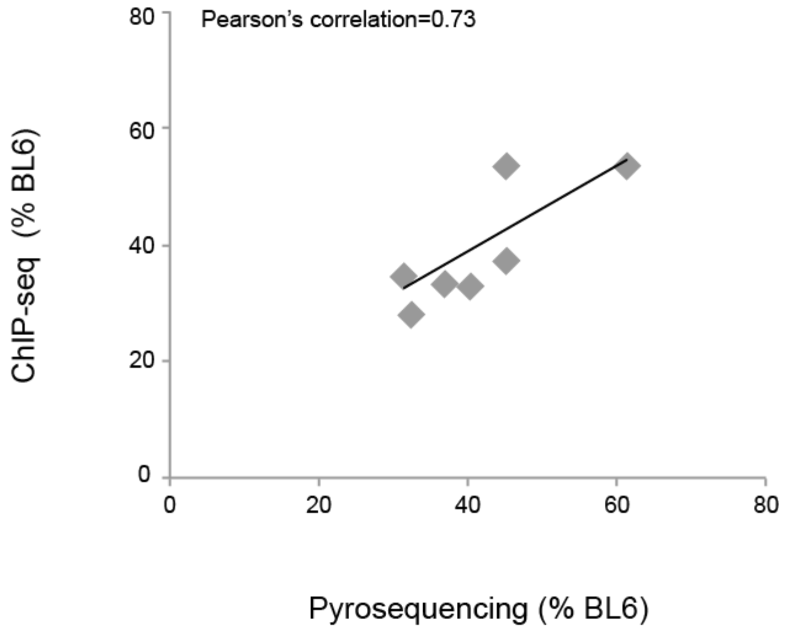

Supplementary Figure 6. Validation of allele-specific measurements made using ChIP-seq. On the y-axis, a measure of allele-specific occupancy change (using the BL6 allele as the reference) determined from ChIP-seq is plotted for seven SNVs, each of which is located under a separate CEBPA peak. On the x-axis, the corresponding measure of allele-specific binding occupancy determined from pyrosequencing is shown. ChIP-seq measurements were derived from the average allelic ratio across all F1 individuals in the study. Pyrosequencing measurements were taken as the average measurement across six biological replicates (averaging across the three technical replicates). Importantly, the correspondence between the ChIP-seq and the pyrosequencing data was high for SNVs even at moderate levels of binding occupancy change in allelic ratio. SNVs for which primer allelic biases were detected were not included in plot (see **Supplementary Table 9** for full list of primers).

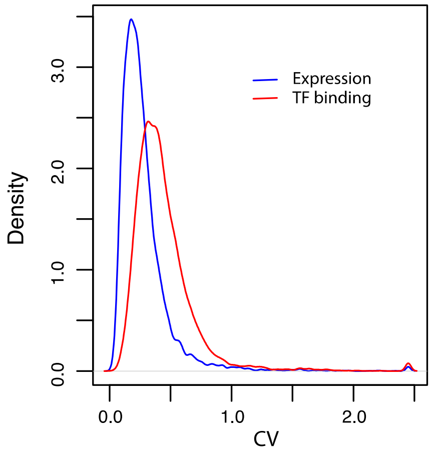

Supplementary Figure 7. TF binding measurements are slightly noisier than gene expression. We use the coefficient of variance (SD/mean), a standardized measure of variance, to compare the level of noise across our replicates for TF binding to that of gene expression. The figure shows that expression measurements are more consistent across replicates than TF binding occupancy measurements. This is not surprising as the expression level of a gene can be estimated by many more SNVs than TF binding (thus reducing residual error). Therefore, the difference between TF binding and gene expression shown in Figure 2A cannot be explained by the difference in experimental error or biological noise.

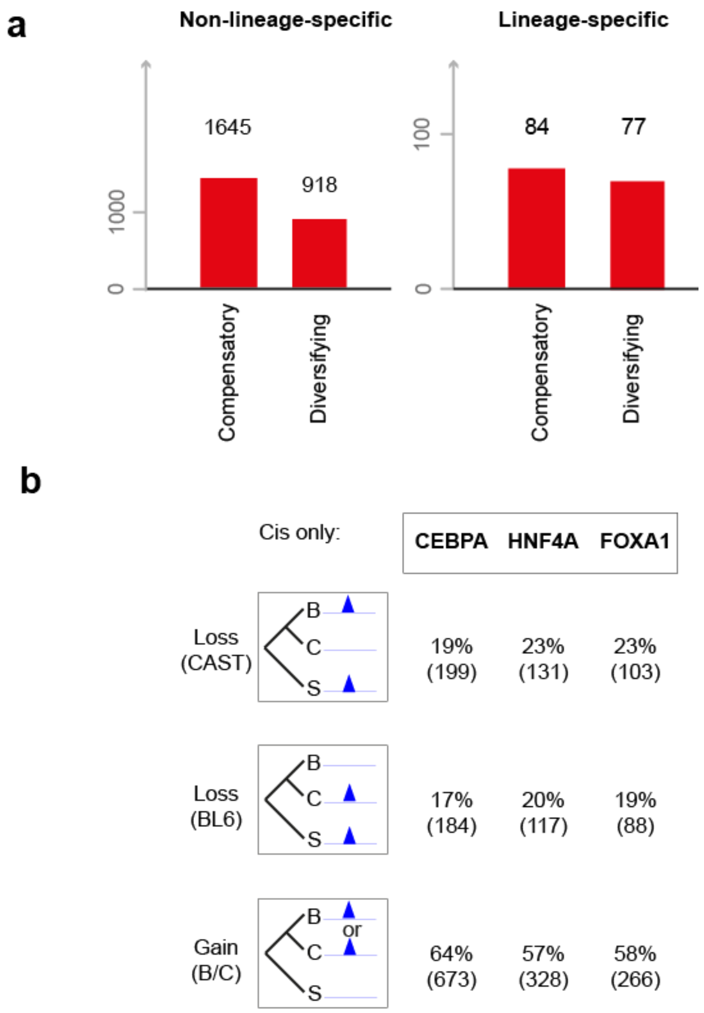

Supplementary Figure 8. Compensatory versus diversifying cistrans modes of regulation. (**A)** Sites influenced by variation in cistrans can be further classified into those showing either diversifying or compensatory effects. These can then be compared to cistrans categorized sites from all non-lineage-specific binding events. Data for CEBPA is shown; data for all factors provided in Supplementary Table 3 **(B)** Highly allele-specific TFBSs affected only by variation in cis were classified into those that were gained in BL6 or CAST and those which were lost in in BL6 or CAST based on parsimony by comparison with TF binding data in *Mus spretus* (counts in brackets).

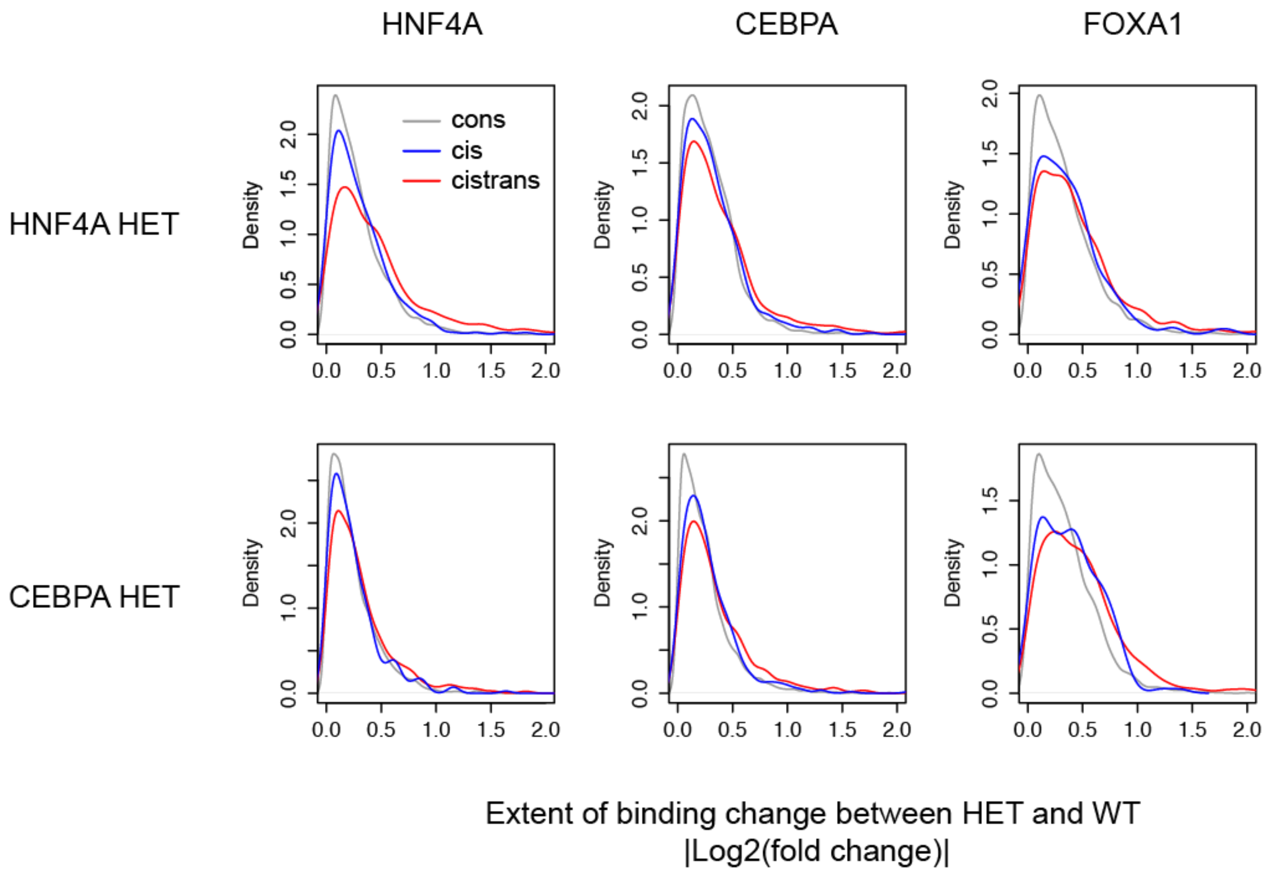

Supplementary Figure 9. Perturbation of TF expression is more likely to cause changes in the binding occupancy of TFBSs influenced by cis and cistrans variation. To restrict analyses to confidently called regulatory categories, only TFBSs assigned cis and cistrans with BIC>2 are shown.

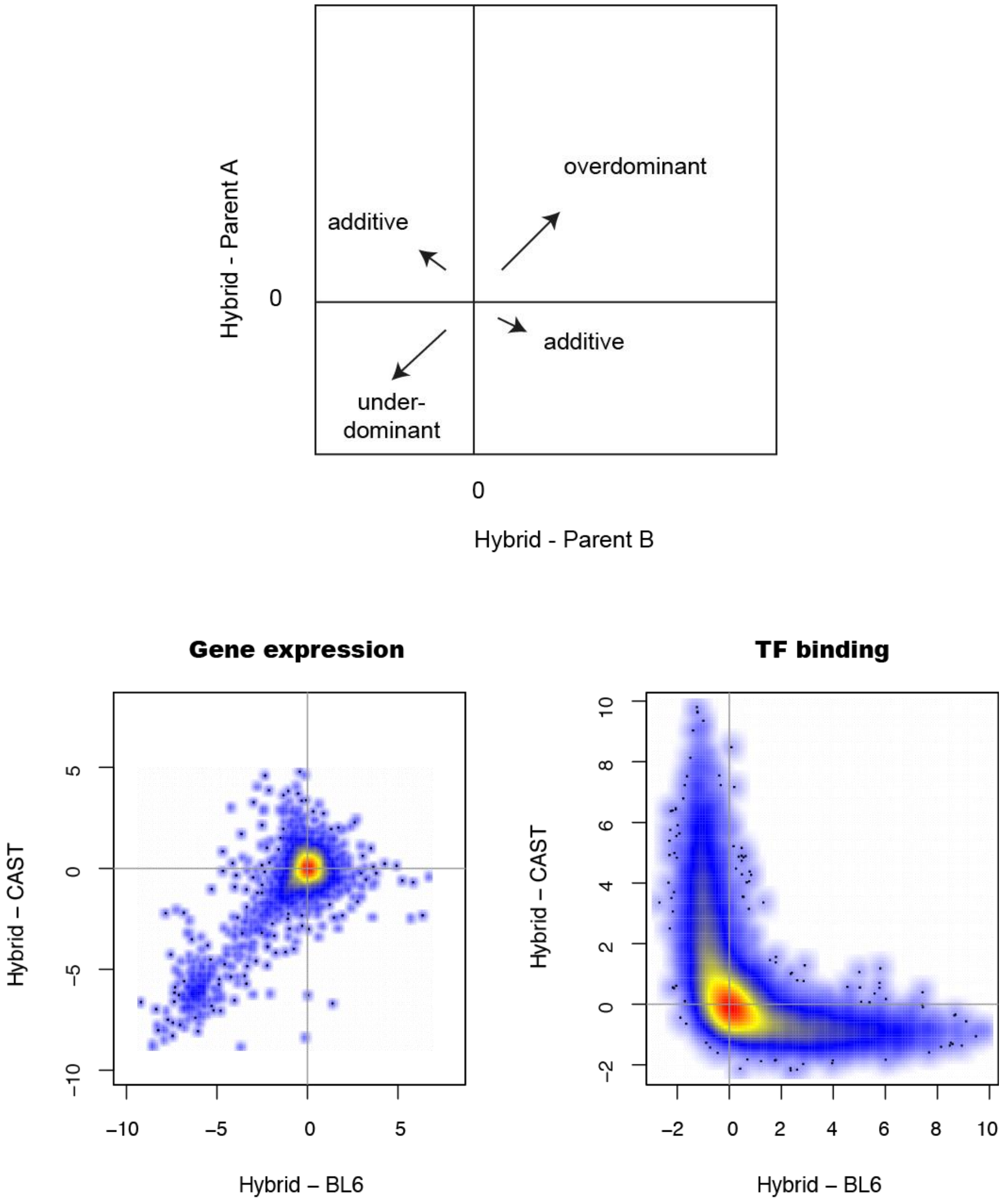

Supplementary Figure 10. Patterns of inheritance of gene expression and TF occupancy levels are distinctly different. Smoothed density scatterplots showing the distribution of inheritance patterns for genes and TFBSs (CEBPA). Hybrid and parental values were summed across both alleles. Axes show log2 transformed parental subtracted from log2 transformed hybrid values (i.e. log2 (hybrid) - log2 (parental)). All values have been normalized for sequencing depth differences across F0 and F1 libraries.

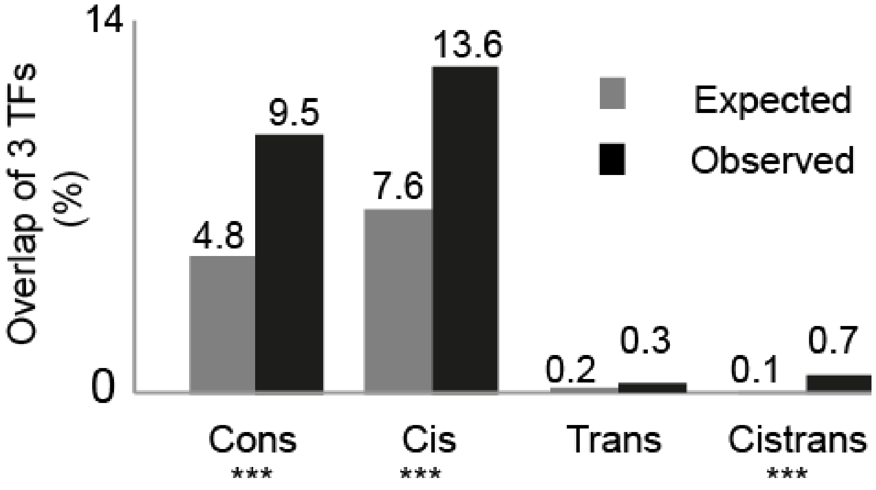

Supplementary Figure 11. Co-bound TFBSs show coordination in their mode of regulation. Percentage of expected and observed instances where all three co-locating TFBSs (CEBPA, HNF4A, FOXA1) are categorized as cis, trans, conserved and cistrans. ***P<0.0001.

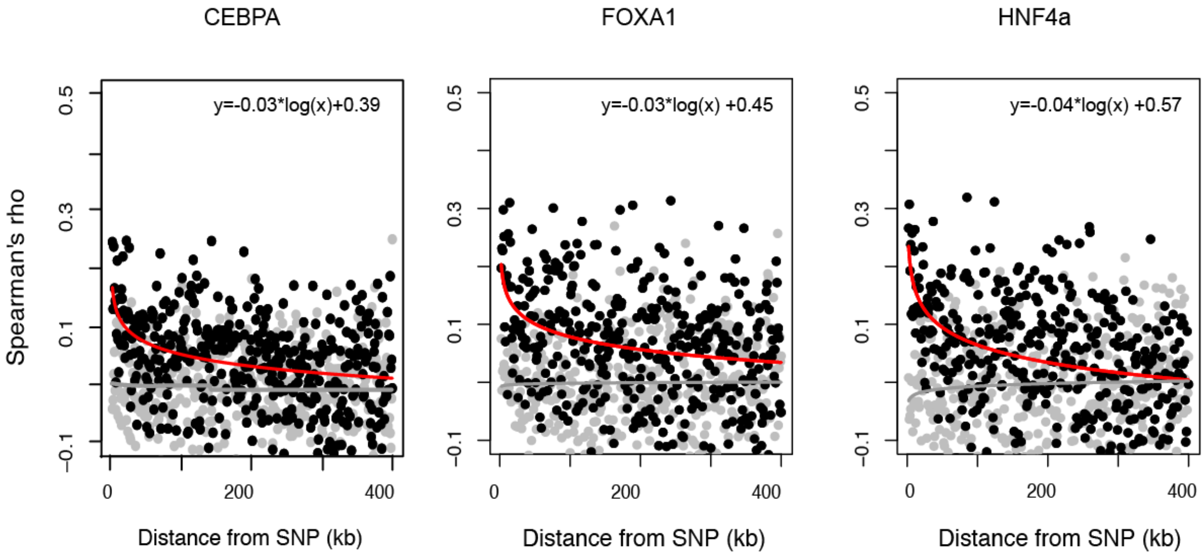

Supplementary Figure 12. Rapid loss of cis-mediated inter-peak correspondence with genomic distance (0-400kb) Spearman's ρ values for each bin were plotted for each TF. Red solid line is the linear regression line. Grey dots represent the background distribution. These data points were constructed by random subsampling of TFBSs to anchor TFBSs (see Methods). The numbers of TFBSs in each randomly sampled bin were matched to those in the observed bins. The grey line is the linear regression line for the correlation values derived from sampled points.

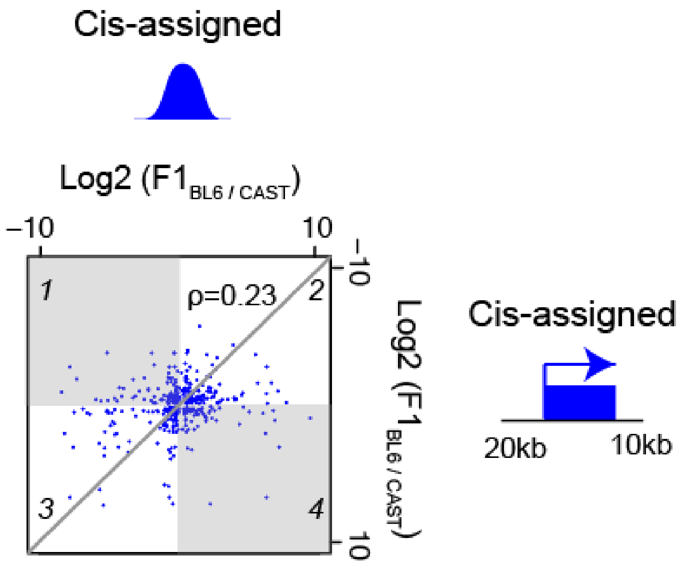

Supplementary Figure 13. Comparison of allele-specific ratio for cis-assigned TF binding intensities and gene expression values for cis-assigned genes. TFBSs were associated to a gene based on their location between 20kb upstream and 10kb downstream of the TSS of an expressed protein-coding gene whose expression variation is influenced by variation in cis. Averaged values across biological replicates are plotted. The probability of points lying in quadrants 2 and 3 is 0.6.

strain	factor	Library ID	raw read #	aligned read #	peak #
BL6	FOXA1	do1071	27,900,124	27,900,124	71,522
		do845	22,693,631	22,693,631	68,857
		do463	20,036,435	20,036,435	66,256
		do3761	42,825,082	34,903,526	86,758
		do3782	20,308,467	15,926,576	55,946
		do3791	36,223,264	28,505,239	70,448
	HNF4A	do562	17,406,988	17,406,988	78,680
		do4129	60,838,932	55,270,985	58,417
		do3781	14,678,134	11,443,041	56,048
		do4293	37,141,421	26,900,486	40,208
		do3864	21880660	16576095	43411
		do3877	51,855,236	38,413,916	42,648
	CEBPA	do560	8,920,790	8,920,790	55,216
		do843	22,741,226	22,741,226	58,368
		do3790	52,879,959	38,034,765	78,468
		do4128	61354397	55,680,196	75542
		do3876	28,266,275	19,007,960	57,286
		do3928	29,677,312	21,812,984	79,346
	H3K4me3	do3339	11,736,386	10,197,385	16,856
		do3340	13,449,788	11,334,199	14,880
		do3341	12,050,060	10,213,004	14,944
CAST	FOXA1	do729	25053520	25053520	76374
		do726	27750358	27750358	80781
		do3794	27057365	20948545	66855
		do3785	34040884	26353127	91589
		do3407	54341317	35561056	57335
		3873	62173712	47796246	89854
	HNF4A	do721	24621464	24621464	83233
		do727	27169999	27169999	82434
		do3784	30968750	23022486	58770
		do3867	17096133	12365382	58961
		do3931	53,336,454	37,812,711	53,600
		do3929	31,592,424	23,724,320	70,139
	CEBPA	do723	28021622	28021622	73770
		do4133	53453510	48824934	66081
		do3783	20429841	28021527	86440
		do4132	59833884	54699714	67517
		do3930	47,870,747	32,323,753	86,387
		do3932	37,529,033	25,543,198	69,059
	H3K4me3	do3342	12729640	10403674	14086
		do3343	12781875	10523978	14063
		do3344	12460927	10066194	13855

Supplementary Table 1. BL6 and CAST F0 experimental data overview

strain	factor	Library ID	raw read #	aligned read #	peak #
BL6xCAST	FOXA1	do1119	34751209	34751209	69274
		do3488	16592875.75	13816534	69156
		do3768	176820540	129691149	65293
		do3778	24537367	18997281	60679
		do3779	18612285	14996168	76257
		do3797	19169934	14742746	55900
	HNF4A	do3483	26571381	22448249	75797
		do1116	37158254	37158254	89664
		do1137	30048282	30048282	67756
		do3769	200228270	147193583	61788
		do3773	22377343	17400623	67601
		do3772	20710280	15878949	71296
	CEBPA	do4134	63252928	58015847	70032
		do4135	59287466	54253843	72698
		do3796	22877754	16642790	77997
		do3777	37341003	27068707	82885
		do3776	15721254	11532802	86205
		do3751	25194339	13789735	91174
	H3K4me3	do3345	13027690	10319854	11415
		do3346	13650567	11078257	12110
		do3347	11959744	10026347	12692
	H3K27ac	do3369	12647503	12336113	27588
		do3370	12442968	12121305	28337
		do3371	13560314	13201976	31449
CASTxBL6	FOXA1	do3515	40,935,141	34,522,412	69,026
		do3519	37,467,446	30,712,508	63,018
		do3521	30,225,016	25,658,264	60,361
		do3524	38,510,080	32,267,180	53,138
		do3892	27,409,387	21,198,132	53,701
		do3896	94,958,248	70,754,403	42,176
	HNF4A	do3462	39,608,271	32,304,528	53,752
		do3937	12,369,819	9,600,374	50,166
		do3934	36,909,737	28,967,267	61,652
		do3935	11,280,829	7,084,060	47664
		do3936	28,165,916	21,357,777	44880
		do3764	16,318,917	15,650,261	42,826
	CEBPA	do3463	31,497,652	23,352,611	72,521
		do3798	25,309,020	18,304,030	64,615
		do3774	23,389,826	15,883,485	73,448
		do3775	24,787,001	16,961,439	65,197
		do3788	29,071,502	21,261,743	72,867
		do1138	31,708,988	31,708,988	52,630
	H3K4me3	do3336	13,151,462	10,889,264	13,812
		do3337	13,804,586	11,603,698	14,698
		do3338	12,734,232	10,656,256	14,209
	H3K27ac	do3360	13994672	13630606	24,278
		do3361	11628345	11361126	24,456
		do3362	12193008	11870803	24,823

Supplementary Table 2. F1 experimental data overview

TF	# cis	# cistrans	# compensatory cistrans	#diversifying cistrans
**CEBPA**	1056	161	84	77
**HNF4A**	606	117	78	34
**FOXA1**	514	77	34	43

Supplementary Table 3. Lineage-specific compensatory versus diversifying cistrans modes of regulation for all factors.

TF	category	Additive (F1 ~= F0b & F1 ~= F0c (MLE))			Dominant (low) (F1 = F0b or F1 = F0c (MLE) where F0=F0(MIN))			Dominant (high) F1 = F0b or F1 = F0c (MLE) where F0=F0(MAX)		
		F0(MAX)=BL6	F0(MAX)=CAST	Total	F0(MAX)=BL6	F0(MAX)=CAST	Total	F0(MAX)=BL6	F0(MAX)=CAST	Total
**FOXA1**	cis (BIC>1, diff_f0>19)	539	599	1138	118	170	288	133	202	335
	trans (BIC>1, diff_f0>19)	31	24	55	59	48	107	27	33	60
	all categories (BIC>1) n=8893									
**CEBPA**	cis (BIC>1, diff_f0>19)	823	897	1720	128	128	256	191	215	406
	trans (BIC>1, diff_f0>19)	20	41	61	59	132	191	52	37	89
	all categories (BIC>1) n=10466									
**HNF4A**	cis (BIC>1, diff_f0>19)	605	647	1252	145	143	288	171	160	330
	trans (BIC>1, diff_f0>19)	14	16	30	36	23	59	16	35	51
	all categories (BIC>1) n=8134									

Supplementary Table 4. Results from additive and dominant inheritance analysis for all factors

TF	category	Over-dominant (F1 ~= F0b & F1 ~= F0c (MLE) & meanf1>meancf0 & meanf1>meanbf0)			Under-dominant (F1 ~= F0b & F1 ~= F0c (MLE) & meanf1<meancf0 & meanf1<meanbf0)		
		F0(MAX)=BL6	F0(MAX)=CAST	Total	F0(MAX)=BL6	F0(MAX)=CAST	Total
**FOXA1**	cis (BIC>1)	9	9	18	0	2	2
	trans (BIC>1)	0	0	0	0	1	1
	all categories (BIC>1) n=8893	20	36	56	49	26	75
	all categories n=61335	142	172	314	266	228	494
**CEBPA**	cis (BIC>1)	2	9	11	1	5	6
	trans (BIC>1)	1	2	3	0	0	0
	all categories (BIC>1) n=10466	25	36	61	14	23	37
**HNF4A**	cis (BIC>1)	3	4	7	2	3	5
	trans (BIC>1)	0	0	0	0	0	0
	all categories (BIC>1) n=8134	20	21	41	19	25	44
**mRNA**	all categories n=11462	632	685	1317	361	325	686

Supplementary Table 5. Results from over- and under-dominant analysis for all factors

TF	Bin size	Analysis from 1kb to 50kb			Analysis from 1kb to 400kb		
		Regression equation	R^2	p-value	Regression equation	R^2	p-value
**CEBPA**	**bin .5kb**	y=-0.05*log(x) +0.55	0.13	1.40E-04			
	**bin 1kb**	y=-0.05*log(x) +0.54	0.21	5.20E-04	y=-0.03*log(x)+0.39	0.12	1.10E-12
	**bin 2kb**	y=-0.05*log(x) +0.56	0.42	2.70E-04			
**FOXA1**	**bin .5kb**	y=-0.05*log(x) +0.60	0.1	1.10E-03			
	**bin 1kb**	y=-0.04*log(x) +0.55	0.13	5.80E-03	y=-0.03*log(x) +0.45	0.1	1.30E-10
	**bin 2kb**	y=-0.04*log(x) +0.49	0.22	9.70E-03			
**HNF4A**	**bin .5kb**	y=-0.05*log(x) +0.67	0.14	1.10E-04			
	**bin 1kb**	y=-0.06*log(x) +0.68	0.27	7.50E-05	y=-0.04*log(x) +0.57	0.16	<2.2e-16
	**bin 2kb**	y=-0.06*log(x) +0.72	0.67	5.80E-07			

Supplementary Table 6. Correspondence between TF binding occupancies. Results are consistent across several bin sizes.

Odds ratio of chromatin contact (relative to Cis)	Cons	Trans	Cistrans
**CEBPA**	**1.20 ***** [1.08-1.34]	1.07 [0.94-1.22]	1.02 [0.90-1.14]
**FOXA1**	**1.16 **** [1.05-1.29]	**1.26 ***** [1.10-1.44]	**0.82 ***** [0.71-0.95]
**HNF4A**	**1.14 *** [1.03-1.27]	1.10 [0.91-1.29]	0.97 [0.84-1.12]

Supplementary Table 7. Odds ratios for chromatin contact enrichment at different regulatory categories. We derived odds ratios from the coefficients of a logistic regression analysis. The underlying regulatory mechanisms were regressed against whether a TFBS location overlapped a region displaying enrichment for long-range chromatin contact (as determined by Hi-C). 95% confident intervals are presented in brackets. ***P<0.0001; **P<0.001; *P<0.05.

Primer name	Sequence	PCR product
**HNF4α ko mice**		
HNF4a_pp2_F	CAGCCCAAGGGAGAGAAGTG	500bp on excised allele
HNF4a_exc_junc8_R	CTGTGAGCCCTGGGAATCAG	
HNF4a_exc_1_F	TACTACCCAGGCTCCCTTCC	129bp on WT allele
HNF4a_exc_1_R	AGTGTGTAGCACAGGGTTCG	
**C/EBPα ko mice**		
6017_F	TGGCCTGGAGACGCAATGA	269bp on targeted, 235bp on WT allele
6072_R	CGCAGAGATTGTGCGTCTTT	
Cebpa_excision_F	GCCTGGTAAGCCTAGCAATCCT	300bp on excised allele
Cebpa_excision_R	TGGAAACTTGGGTTGGGTGT	

Supplementary Table 8. Primers used for the genotyping of heterozygous knockout mice

Primer name	Forward primer	Reverse primer	Sequencing primer
**cis_1**	TAAGCTGCGAGAACCTCTGAT	[Btn]CCTGCTTGGCTTGTGTGAAT	ACGTCCTCCCTGACC
**cis_2**	[Btn]ACTGGCAAGAAGCAATGAGC	TCTCATGGGGGACTTCGG	GTAGGGCCTGGGCGT
**cis_3**	TATGGGGATTACGGGGTCTG	[Btn]GTTTGGAAAGAACCCCGACAG	GAAAGTGGAAAGCCTC
**cistrans_1**	AAAGGGAGCCTGGAACCACAT	[Btn]GGCATCCATCTTGACAGGAGTT	GAACCACATCTGCCTC
**cistrans_2**	[Btn]TGCAAGGAGCCATCATTCT	CACATCCGTTTGTTGCTGAG	ACTGAACTACATACTTACCA
**cistrans_3**	AGCTACTCTGAAGCGGTTTGC	[Btn]CCTGGGGCTTCACATCAAT	GAGGAAAAAAGAAATGTAGA
**conserved_1**	[Btn]TGTGTGTGCTGCAACTGATGG	GGGAGGCTTAGGAAGAGGTCAATA	CGGGGAGGAGGTGTG
**trans_1**	ATGCTTTGAACTGTTGCACTGTCT	[Btn]CCCCTAAGCAAGTCTCAAAGTG	TCTCTTCAGCTCAATTCTC
**trans_2**	CTGCTTGGTGCCTGTGCT	[Btn]GGTAACCAGAGTAGCGGCTCAG	CTGCGACCGAGCCAG
**trans_3**	GCTGGCAAGTGACCCTGAGT	[btn]AGTCCCTGAACAGACACCCACTTA	CAGTATAGTTAGGAATCCCC

Supplementary Table 9. Primers used for pyrosequencing validation
